# Identification of oxidized protein hydrolase as a potential prodrug target in prostate cancer

**DOI:** 10.1186/1471-2407-14-77

**Published:** 2014-02-10

**Authors:** Christopher A McGoldrick, Yu-Lin Jiang, Victor Paromov, Marianne Brannon, Koyamangalath Krishnan, William L Stone

**Affiliations:** 1 Department of Pediatrics, East Tennessee State University, P.O. Box 70579, Johnson City, TN 37614, USA; 2 Department of Chemistry, East Tennessee State University, Johnson City, TN USA; 3 Division of Hematology-Oncology, Department of Internal Medicine, East Tennessee State University, Johnson City, TN USA

## Abstract

**Background:**

Esterases are often overexpressed in cancer cells and can have chiral specificities different from that of the corresponding normal tissues. For this reason, ester prodrugs could be a promising approach in chemotherapy. In this study, we focused on the identification and characterization of differentially expressed esterases between non-tumorigenic and tumorigenic prostate epithelial cells.

**Methods:**

Cellular lysates from LNCaP, DU 145, and PC3 prostate cancer cell lines, tumorigenic RWPE-2 prostate epithelial cells, and non-tumorigenic RWPE-1 prostate epithelial cells were separated by native polyacrylamide gel electrophoresis (n-PAGE) and the esterase activity bands visualized using α-naphthyl acetate or α-naphthyl-N-acetylalaninate (ANAA) chiral esters and Fast Blue RR salt. The esterases were identified using nanospray LC/MS-MS tandem mass spectrometry and confirmed by Western blotting, native electroblotting, inhibition assays, and activity towards a known specific substrate. The serine protease/esterase oxidized protein hydrolase (OPH) was overexpressed in COS-7 cells to verify our results.

**Results:**

The major esterase observed with the ANAA substrates within the n-PAGE activity bands was identified as OPH. OPH (EC 3.4.19.1) is a serine protease/esterase and a member of the prolyl oligopeptidase family. We found that LNCaP lysates contained approximately 40% more OPH compared to RWPE-1 lysates. RWPE-2, DU145 and PC3 cell lysates had similar levels of OPH activity. OPH within all of the cell lysates tested had a chiral preference for the S-isomer of ANAA. LNCaP cells were stained more intensely with ANAA substrates than RWPE-1 cells and COS-7 cells overexpressing OPH were found to have a higher activity towards the ANAA and AcApNA than parent COS-7 cells.

**Conclusions:**

These data suggest that prodrug derivatives of ANAA and AcApNA could have potential as chemotherapeutic agents for the treatment of prostate cancer tumors that overexpress OPH.

## Background

Prostate cancer is the second most frequently diagnosed cancer in men and the second-leading cause of cancer related death in American men [1]. There is an estimated 238,590 new cases of prostate cancer predicted in the US this year and an estimated 29,720 deaths due to prostate cancer [1]. Despite advances in radiation and chemotherapy, prostate cancer is a leading cause of cancer death. Radiation and chemotherapy treatment remain central to prostate cancer treatment. These treatments can, however, produce a number of side effects such as neutropenia [2,3], urinary and bowel symptoms [4], hair loss [5], and fatigue [6]. There is, therefore, a critical need to develop tumor specific therapies for prostate cancer.

**Figure 1:**
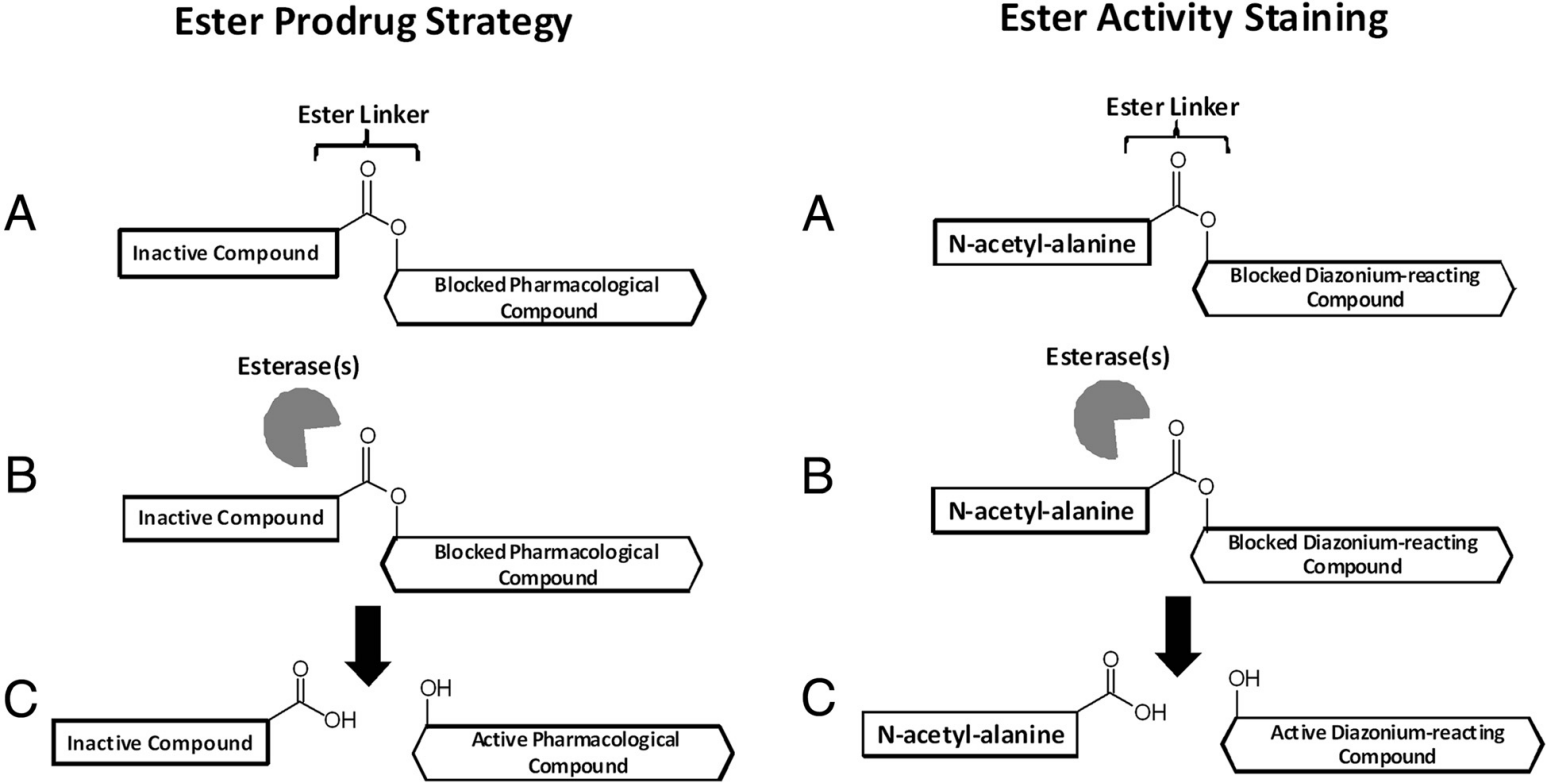
**Esterase activity profiling and the esterase prodrug strategy proposed by Yamazaki et al. utilize the same mechanism for activation. A)** The active compound is blocked with an ester linker to an inactive compound such as N-acetyl-alanine. **B)** The compound is activated within the target cell by target esterase(s). **C)** The prodrug is unblocked and induces cell death in the target cell. Esterase activity staining with ANAA substrates releases naphthyl alcohol upon hydrolysis that reacts with Fast Blue RR, a diazonium salt, to form an insoluble product.

Selective activation of anti-cancer drugs within cancer cells is a promising strategy to minimize the toxic effects of anticancer drugs on normal tissues [7-10]. As indicated in Figure 1, the esterase prodrug strategy utilizes pharmacological compounds that are blocked by esterification but are activated when cancer cell esterases cleave the ester bond and release the active drug [11]. A degree of specificity can be achieved if the cancer cell esterase is overexpressed compared to normal tissue. In order to optimize potential chemotherapeutic prodrug esters it is important to characterize and identify any differentially expressed esterases.

**Figure 2:**
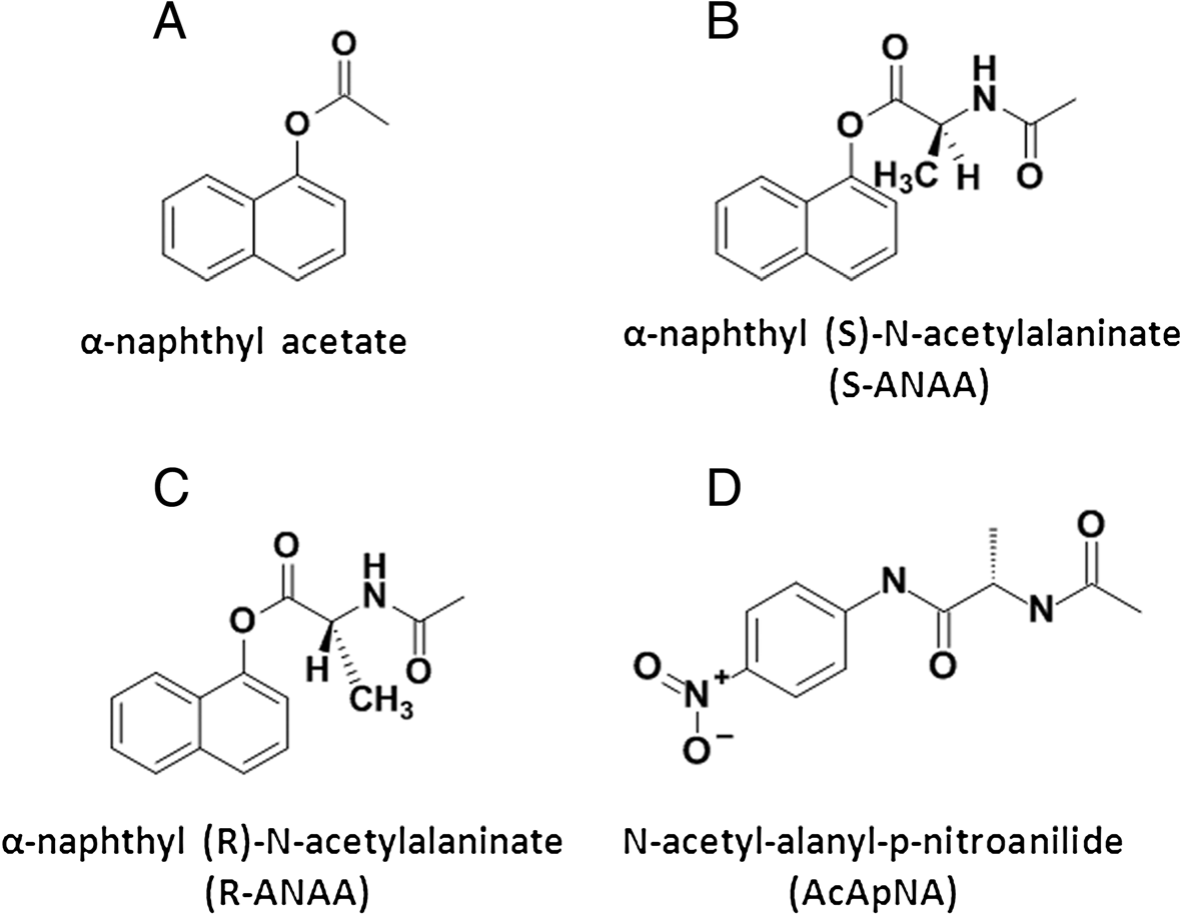
**Structures of compounds used to evaluate esterase activity profiles. A)** α-naphthyl acetate is a non-specific esterase substrate and is used to visualize general esterase activity. **B)** S-ANAA and **C)** R-ANAA are chiral esters previously used by Yamazaki et al. to demonstrate stereoselective preferences between cancer and normal cells. **D)** N-acetyl-L-alanyl-p-nitroanilide releases p-nitroanaline upon hydrolysis and is routinely used to monitor OPH activity.

Yamazaki et al. [12-14] examined the esterase activity profiles of various human and animal cancer tumors using n-PAGE and esterase activity staining. These researchers found that lysates from cancer tumors often had a different level of activity and a different stereoselectivity towards several chiral esters than the corresponding normal tissues. Moreover, Yamazaki et al. suggested these differences in esterase activities could be exploited to develop prodrugs that selectively target cancer cells [13,14]. The esterases observed by Yamazaki et al. [12-14] were, however, never identified. The primary focus of the work presented here was to identify the specific esterases differentially expressed in tumorigenic human prostate cancer cells and in non-tumorigenic prostate epithelial cells. We compared the esterase activity profiles of RWPE-2, LNCaP, DU 145, and PC-3 tumorigenic prostate cell lines to RWPE-1 nontumorigenic prostate epithelial cells using the α-naphthyl acetate substrate and the chiral naphthyl ester substrates α-naphthyl N-acetyl-S-alaninate (S-ANAA) and α-naphthyl N-acetyl-R-alaninate (R-ANAA). These substrates were previously used by Yamazaki et al. [13]. Figure 2 shows the structures of the various substrates. In addition, we have advanced the Yamazaki method of detecting esterases by using a native electroblot method that markedly increases the sensitivity for detecting esterase activity bands compared to that observed in n-PAGE gels.

We identified oxidized protein hydrolase (OPH), also called N-acylaminoacyl-peptide hydrolase (APEH), as a key esterase that is overexpressed in the tumorigenic LNCaP cell line. OPH is a serine esterase/protease that has a well characterized esterase activity towards α-naphthyl butyrate [15] and an exopeptidase activity for removing the N-terminally acetylated amino acid residues from peptides/proteins [15-17]. Immunohistochemistry of primary prostate tumor sections indicate that OPH is highly expressed in some prostate tumors (http://www.proteinatlas.org/), suggesting that OPH could have potential as a drug target in prostate cancer. The overexpression of OPH in some prostate cancers suggests that chemotherapeutic prodrugs esters modeled after known ester substrates of OPH (i.e., α-naphthyl N-acetyl-alaninate) have potential in treating some prostate cancers.

## Methods

### Materials

Porcine liver esterase (PLE), digitonin, α-naphthyl acetate, fast blue RR salt, goat anti-rabbit HRP conjugate polyclonal antibody, and diisopropyl fluorophosphate (DFP) were purchased from Sigma Chemical Company (St. Louis, MO). Novex Tris-glycine native sample buffer, NuPAGE LDS sample buffer, Novex Tris-glycine gels, NativeMark unstained protein standards, Protein A agarose beads, penicillin/streptomycin solution, and geneticin (G418) were purchased from Invitrogen (Grand Island, NY). Precision plus protein standards were purchased from Bio-Rad (Hercules,CA); the BCA kit and the In-gel tryptic digestion kit were purchased from Pierce (Rockford, IL); ZipTipU-C18 tips were purchased from Millipore (Billerica, MA); 3,3′,5,5′-tetramethylbenzidine (TMB) was purchased from Promega (Madison, WI); rabbit polyclonal anti-AARE antibody was purchased from Abcam (Cambridge, MA); superose 12 column (10/300 GL) was purchased from GE Healthcare (Pittsburgh, PA); (TransIT-LT1 transfection reagent was purchased from Mirus Bio (Madison, WI). pCDNA3.1(+) vector encoding OPH-Flag was a kind gift from Dr. M. Hayakawa (Tokyo University of Pharmacy and Life Sciences, Tokyo, Japan).

### Substrates

R- and S- isomers of ANAA (Figure 2) were synthesized and purified as previously described [13] and stored at -20°C. Stock solutions of 100 mM α-naphthyl acetate were prepared in DMSO and stored at -20°C. The synthesis of N-acetyl-alanyl-p-nitroanilide (AcApNA) was guided by a previously published procedure [18]. AcApNA was synthesized by adding 20 ml of dichloromethane to a solution of anhydrous dimethylformamide (0.51 ml) and 0.865 g of N-acetyl-L-alanine. The mixture was cooled to -20°C with an acetone-dry ice bath. Thionyl chloride (0.485 ml) was added dropwise to the cooled mixture. After 20 min, a cold solution (-20°C) of 0.828 g 4-nitroanaline and 1.82 ml of triethylamine in 10 ml of dichloromethane was added dropwise to the N-acetyl-L-alanine solution. The resulting mixture was maintained at 0°C for 2 h. After concentration, the mixture was extracted with ethyl acetate (2 × 30 ml). The organic layer was washed with 4 N HCl (2 × 40 ml) and NH_4_Cl aqueous solution (40 ml), and dried over MgSO_4_. After filtration and concentration of the organic layer, the residue was purified using column chromatography with hexane, then 30-50% acetone in hexane, affording 0.248 g of the final product (16%). M.P. was found to be 194-196°C. The M.P. has been previously reported as 192-197°C [19]).

### Cell culture and lysates

RWPE-1 (CRL-11609), RWPE-2 (CRL-11610), LNCaP (CRL-1704), DU-145 (HTB-81), PC-3 (CRL-1435) and COS-7 (CRL-1651) cell lines were purchased from American Type Culture Collection (Manassas, VA), cultured according to ATCC’s instructions and supplemented with 100 U/ml penicillin and 100 mg/ml streptomycin. Cells were detached from the 75 cm^3^ cell culture flasks after reaching 80% confluence by washing the cells with PBS followed by the addition of 0.25% trypsin. The detached cells were centrifuged at 500 × g for 5 mins and washed with PBS to remove trypsin. Cells were centrifuged a second time and pellets stored at -80°C. Cell pellets of each cell line were lysed using 2% (wt/vol) digitonin in PBS on ice with vortexing every two minutes. After 10 min of incubation on ice, the lysates were centrifuged at 18,000 × g for 5 min at 4°C and the supernatant collected. Protein concentrations were determined with the BCA kit using the manufacturer’s instructions.

### n-PAGE esterase activity profiles

Cell lysates containing 120 μg of protein were mixed with an equal volume of 2X Novex Tris-glycine native sample buffer and applied to a Novex 10-20% or 6% Tris-glycine gel. NativeMark unstained protein standards were used as migration markers. Gels were electrophoresed under native conditions at 4°C using 20 mA/gel for 270 min for the 10-20% gel or 180 min for the 6% gels. For inhibition assays, the gel lanes were separated and immersed in either 0.1 M sodium phosphate buffer, pH 6.5 or sodium phosphate buffer containing 50 μM DFP for 10 min. The gels were then stained for esterase activity by immersing them in 30 ml of 0.1 M sodium phosphate buffer, pH 6.5 containing 10 mg Fast Blue RR Salt and 800 μM α-naphthyl acetate or 800 μM ANAA isomer. Bands were developed at room temperature for 30 min followed by 3 washes with distilled water. The migration markers were stained with Coomassie blue and destained overnight in 10% acetic acid. Gels were scanned with an Epson Perfection V750 PRO scanner.

### Semi-purified OPH from rat liver

OPH was semi-purified from 100 g of rat liver using the method described by Tsunasawa [20] with the following modifications. After elution from the hydroxyapatite column, the OPH fractions were combined and subjected to gel filtration on a Superose12 column (10/300 GL) using a Biologic Duo Flow protein purification system (Bio-Rad, Hercules,CA). Fractions were eluted with 50 mM sodium phosphate buffer, pH 7 containing 1 mM EDTA and 0.2 M NaCl at a rate of 0.5 ml/min in 0.5 ml fractions. Fractions that contained OPH activity were combined and stored at -20°C. The pooled semi-purified OPH was analyzed by mass spectroscopy to verify that no other esterases or proteases were present.

### Overexpression of OPH in COS-7 cells

COS-7 cells were transfected using TransIT-LT1 transfection reagent and the vector pCDNA3.1(+) encoding OPH with a Flag tag using the transfection reagent’s manufacturer’s instructions. COS-7 cells overexpressing OPH were selected using 1 mg/ml G418 over a three week period. Cells surviving selection were termed COS-7-OPH for further experiments and were maintained with 1 mg/ml G418.

### LC/MS-MS mass spectroscopy

Protein bands were individually excised from the n-PAGE gel and cut into small pieces using a scalpel. The gel pieces were destained, disulfide bonds reduced, unmodified thiol groups alkylated, and the proteins digested with trypsin overnight using the In-Gel Tryptic Digestion Kit (Pierce, Rockford, IL) according to the manufacturer’s instructions. After digestion, the liquid containing the peptides from each band was transferred to a 1.5 ml tube. The peptides were further extracted from each gel piece by covering gel piece with extraction buffer consisting of formic acid/acetonitrile/water (5:50:45, v/v/v) for 10 min then collecting the liquid and adding it to the appropriate 1.5 ml tube. The peptides in the vial inserts were completely dried using a DNA Speed Vac Concentrator (Thermo Fisher Scientific, Asheville, N.C.). Peptides were rehydrated with 0.1% formic acid and further purified using ZipTipU-C18 tips according to manufacturer’s instructions. Peptides eluted from zip tips were transferred to vial inserts. The peptides in the vial inserts were completely dried using the Speed Vac Concentrator and then rehydrated in a volume of 4 μl of formic acid/acetonitrile/water (0.1:20:79.9, v/v/v). A volume of 2 μl of each sample was trapped by a picofrit column packed with C18 (New Objective, Inc., Woburn, MA) and equilibrated in 0.1% formic acid in water/acetonitrile (98:2, v/v). Peptides were then eluted with a gradient of 2 to 40% of solvent B containing 0.1% formic acid in acetonitrile over 60 min at a flow rate of 200 nL/min. Eluted peptides were analyzed by electrospray ionization using a LTQ-XL ion trap mass spectrometer (Thermo Fisher). Mass spectrometry (MS) data were acquired using data-dependent acquisition with a series of one full scan followed by a zoom scan and then a MS/MS scan of the ions. The dynamic exclusion duration was 30 ms. Proteins were identified from each MS raw data file using the SEQUEST search algorithm (Thermo Fisher Scientific) and the SwissProt/UNIPROT database through the Bioworks browser, version 3.3.

### SDS-electrophoresis/Western blotting

Cell lysates containing 90 μg of protein were mixed with NuPAGE LDS Sample Buffer, heated to 90°C, and applied to a Novex 10-20% Tris-Glycine gel. Precision Plus Protein Standards were used for molecular weight markers. Gels were electrophoresed for 90 min at 125 V in 1X Novex Tris-Glycine SDS running buffer. Gels were then electrophoretically transferred to a nitrocellulose membrane for 90 min at 25 V. The membrane was probed with 1:1000 rabbit polyclonal anti-AARE (OPH) antibody (ab84694, Abcam) or anti-GAPDH (ab9485, Abcam) overnight at 4°C, 1:2000 anti-rabbit IgG conjugated to HRP (A0545-1ML, Sigma) was used as the secondary antibody and incubated for 1 hour. Membranes were washed with PBS containing 0.05% Tween 20. Peroxidase was detected using 3,3′,5,5′-tetramethylbenzidine according to manufacturer’s instructions.

### Native electroblot activity staining and western blotting

Native electroblot activity staining was carried out by electrophoretically transferring proteins from n-PAGE gels to a nitrocellulose membrane at 4°C, followed by the esterase activity staining procedure (see above). Western blots of the n-PAGE gel were carried out by probing a native electroblot as described in the Western blotting methods.

### OPH-cleared lysate

An aliquot of 0.5 ml Protein A agarose beads was coupled with 5 μg of anti-OPH antibody on ice for 30 min. LNCaP cell lysates containing 120 μg of protein was combined with either Protein A agarose beads or anti-OPH conjugated Protein A agarose beads and incubated on ice for 1 hour with gentle mixing every 15 min. The samples were centrifuged for 5 min at 1000 × g and the supernatants were then separated using 6% n-PAGE followed by the esterase activity staining procedure.

### OPH activity assay

Aliquots of 20 μL of cell lysates containing either 4.5 μg/μl of protein, or 0.5 unit PLE, or 12.5 ng/μl semi-purified rat liver OPH, or PBS were added in triplicate to a 96 well microplate. One unit of PLE is defined as the amount of PLE that will hydrolyze 1.0 μmole of ethyl butyrate to butyric acid and ethanol per min at pH 8.0 at 25°C. An assay cocktail of 220 μL 0.1 M sodium phosphate buffer, pH 6.5 and 10 μl of 100 mM AcApNA was added to each well giving a final AcApNA concentration of 4 mM. The release of p-nitroaniline was monitored with a microplate reader at a wavelength of 405 nm for 10 min at room temperature. The concentration of p-nitroaniline was calculated using a molecular extinction coefficient of 7530 M^-1^ cm^-1^.

### Cell culture esterase staining

LNCaP, RWPE-1, COS-7, and COS-7-OPH were seeded in triplicate in 24 well cell culture plates at 1x10^5^ cells/well. The plate was incubated at 37°C in a CO^2^ incubator overnight. Staining solutions of 0.1 M sodium phosphate buffer, pH 6.5 containing 10 mg Fast Blue RR Salt and 800 μM α-naphthyl acetate or 800 μM α-naphthyl N-acetylalaninate isomer were prepared immediately prior to cell staining. The cell media was removed from each well and 500 μl of staining solution was added to each well. The cells were incubated at 37°C in 5% CO_2_ for 20 minutes. The staining solution was removed and replaced with 500 μl of PBS. The cells were observed at 100x magnification and digitally photographed using a MOTIC inverted phase contrast microscope equipped with a Nikon Coolpix E4300 4-megapixel camera (Martin Microscope, Easley, SC). The percent area threshold of staining was measured using ImageJ, v1.44o (NIH, Bethesda, MD).

### Statistics

Data were analyzed by analysis of variance (ANOVA) followed with the Scheffe test for significance with P < 0.05 using SPSS 19.0 for Windows (Chicago, Illinois). Results were expressed as the mean ± SD of at least three experiments. In all figures, letters that are not the same are significantly different with P < 0.05.

### Ethics

The research conducted in this study adhered to US NIH ethical guidelines. All the human cell lines studied were purchased from the American Type Culture Collection and such studies are not considered human subjects research because the cell lines are publicly available and all of the information known about the cell lines is also publicly available. No experimental animals were used in the studies reported here.

## Results

### Differential esterase activity between non-tumorigenic RWPE-1 and tumorigenic LNCaP cells

Our first objective was to determine if non-tumorigenic prostate cells have a different n-PAGE esterase activity profile compared to tumorigenic prostate cells and to characterize any chiral ester substrate preferences.

**Figure 3:**
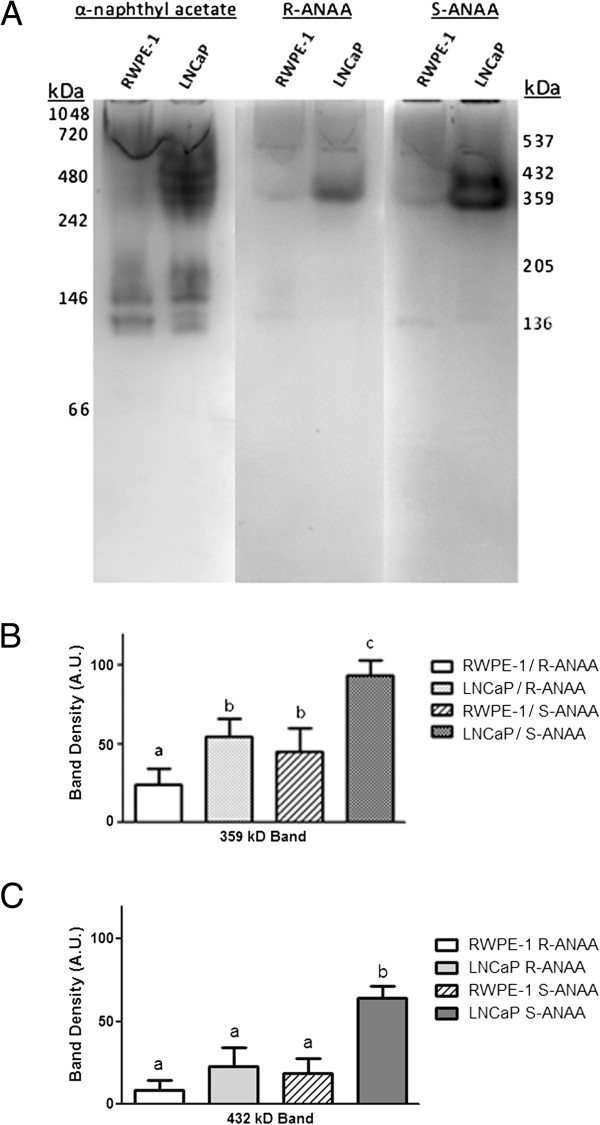
**Non-tumorigenic RWPE-1 and tumorigenic LNCaP prostate cell lysates display differential esterase activity and substrate specificity. A)** Non-tumorigenic RWPE-1 and tumorigenic LNCaP cell lysates containing 120 μg of protein were separated on a native 10-20% polyacrylamide gel. The gels were stained with 800 μM α-naphthyl acetate, R-ANAA, or S-ANAA substrate and Fast Blue RR salt. Native molecular weight markers (left side) were used to estimate the relative migration of some esterase bands (right side). **B)** The 359 kDa n-PAGE LNCaP and RWPE-1 esterase bands stained with R-ANAA or S-ANAA were measured by densitometry. **C)** The 432 kDa n-PAGE LNCaP and RWPE-1 esterase bands were also measured by densitometry. Letters that are not the same are significantly different at P < 0.05.

Proteins from non-tumorigenic RWPE-1 and tumorigenic LNCaP human prostate cell lysates were separated by n-PAGE on a 10-20% gradient gel and stained for esterase activity (Figure 3A) using either α-naphthyl acetate, R-ANAA, or S-ANAA substrates (Figure 2A-C) and Fast Blue RR salt. General esterase activity, as visualized by α-naphthyl acetate activity staining [21,22], was markedly higher in the tumorigenic LNCaP lysate compared to the non-tumorigenic RWPE-1 lysate. Parallel gels stained with either R-ANAA or S-ANAA substrates revealed fewer esterase bands than with α-naphthyl acetate. The chiral substrates revealed two prominent bands that migrated at native protein molecular weight markers locations corresponding to “432 kDa” and “359 kDa”. Protein migration in n-PAGE electrophoresis is influenced by size, conformation and charge and, therefore, the “native kDa markers” in Figure 3A were used only to provide a reproducible measure of electrophoretic migration patterns rather than a meaningful measure of true molecular weight. As shown in Figure 3A, both the “432 kDa” and the “359 kDa” bands were markedly more stained in the LNCaP cell lysates compared to the RWPE-1 cell lysates and both bands showed higher staining with S-ANAA compared to the R-ANAA chiral substrate. Densitometry analysis of the 432 kDa and 359 kDa esterase bands (Figure 3B-C) showed approximately a 30% increase in activity with S-ANAA compared to R-ANAA and approximately 40% more activity with LNCaP lysates compared to RWPE-1 lysates.

### Prostate esterases identified as OPH have a preference for S-ANAA

**Figure 4:**
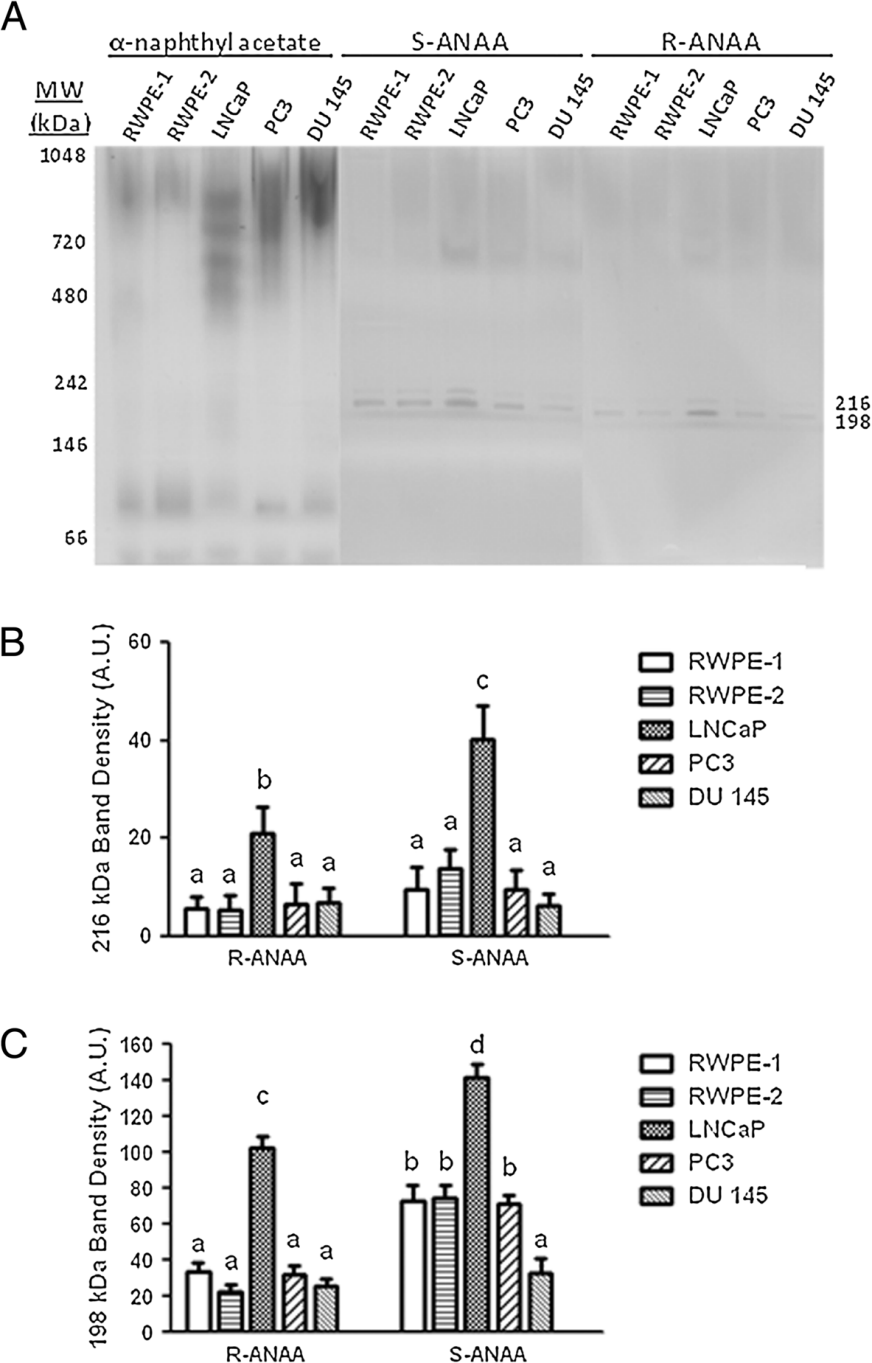
**Prostate cell lysate esterases form distinct bands when separated by 6% n-PAGE. A)** RWPE-1, RWPE-2, LNCaP, DU 145, and PC3 cell lysates containing 120 μg of protein were separated by 6% n-PAGE followed by staining with either 800 μM α-naphthyl acetate, S-ANAA, or R-ANAA. Native molecular weight markers (left side) were used to calculate the relative migration of some esterase bands (right side). The **B)** 216 kDa and **C)** 198 kDa esterase bands visualized with S-ANAA were measured by densitometry. Letters that are not the same are significantly different at P < 0.05.

Initial attempts to identify the esterases within the “432 kDa” and “359 kDa” bands by LC/MS-MS were hindered by the large number of non-esterase proteins. To further characterize esterase activity in human prostate cells, we next examined cell lysates of several human prostate cell lines for esterase activity using 6% n-PAGE followed by activity staining with α-naphthyl acetate, or chiral ester substrates R-ANAA, or S-ANAA (Figure 4A). By performing 6% n-PAGE electrophoresis, the higher MW proteins were better separated and esterase bands generally more defined. The n-PAGE esterase activity profiles obtained with α-naphthyl acetate showed diffuse bands in the 720 to 1048 kDa native protein marker range for LNCaP, DU145 and PC3 cell lysates that were faintly present in the RWPE-1 or RWPE-2 cell lysates. The staining intensity with α-naphthyl acetate in the 720 to 1048 kDa region was greater for the LNCaP, DU145 and PC3 cell lysates compared to the RWPE-1 or RWPE-2 cell lysates.

Parallel gels stained with S-ANAA or R-ANAA show two prominent and sharp esterase bands at 216 kDa and 198 kDa native protein marker points. Densitometry analysis of the 216 kDa band showed significantly higher esterase activity in the LNCaP cell lysate stained with R-ANAA and S-ANAA compared to all other cell lysates (Figure 4B). Moreover, the 216 kDa band for LNCaP cells showed higher activity with the S-ANAA substrate compared to the R-ANAA substrate. The degree of chiral substrate selectivity was more apparent in the esterase activity of the 198 kDa band (Figure 4C). Densitometry of the 198 kDa band showed a significant preference for S-ANAA substrate in all of the cell lines except DU 145. The LNCaP lysates contained 40-50% higher esterase activity in both bands with S-ANAA substrate than with the R-isomer and a 40-60% higher activity compared to RWPE-1 lysate. RWPE-2 and PC3 lysates had similar staining profiles to RWPE-1, while DU 145 showed less activity compared to RWPE-1.

The n-PAGE esterase profiles obtained with S- or R-AANA showed fewer and more distinct bands than with the α-naphthyl acetate. We, therefore, focused on determining the identity of the protein(s) in the more active 198 kDa band. This band was excised, trypsinized, and the resulting peptides were subjected to mass spectrometry analysis to identify the esterase(s) responsible for the activity. As shown in Table 1, we identified oxidized protein hydrolase (OPH), also called N-acylaminoacyl-peptide hydrolase, or acylamino-acid-releasing enzyme (EC 3.4.19.1) in the 198 kDa band. OPH is a serine esterase/protease with a well characterized exopeptidase activity for removing N-terminally acetylated residues from peptides [15-17]. These LC-MS/MS results as well as the hydrolysis of the ANAA substrates by the 198 kDa band are consistent with the known activity of OPH to remove N-terminally acetylated alanine residues.

**Table 1:** LC/MS-MS analysis of 198 kDa n-PAGE bands

**Protein Identical**	**P**	**MW(Da)**
N-acylaminoacyl-peptide hydrolase (OPH)	3.116E-08	81172.77
**OPH Peptides Indentified**	**P**	**MW(Da)**
R.LLLYPK.S	4.144E-01	136.96
K.TPLLLMLGQEDR.R	3.128E-07	440.43
R.SALYYVDLIGGK.C	1.171E-07	1180.98
K.STHALSE VE VE SDSFMNAVLVVLR.T	1.159E-01	213.91
KVGFLP SAGK.E	5.348E-01	280.19
K.ALDVSASDDEIAR.L	2.568E-07	1461.57
K.VTSVVVDVVPR.Q	1.285E-04	966.14
K.SFNLSALEK.H	2.434E-03	512.27
K.QFLEVWEK.N	2.718E-04	201.26
R.GSTGFGQDSILSLPGNVGHQDVK.D	4.639E-02	266.86
K.MGFAVLLVNYR.G	1.412E-01	1143.36
R.SALYYVDLIGGK.C	5.262E-01	567.34
K.GDQFVFYEDWGENMVSK.S	7.935E-02	195.47
R.QDLFAVDTQVGTVTSLTAGGSGGSWK.L	3.116E-08	323.53
K.EQSVLWVSLEEAEPIPDIHWGIR.V	8.643E-02	382.15

A representative list of OPH peptide identified within the 198 kDa esterase band by mass spectrometry. Peptide coverage was 24.6% of the full OPH protein sequence. P indicate the probability that the protein or peptide is a random match to the spectral data.

LNCaP lysates showed significantly higher levels of esterase activity with α-naphthyl acetate and ANAA substrates compared to non-tumorigenic RWPE-1 and tumorigenic RWPE-2, DU145 and PC3 cell lysates. It appears clear that not all tumorigenic prostate cells contain high levels of OPH activity; however, the human protein atlas (http://www.proteinatlas.org/) indicates that some human tumors overexpress OPH compared to normal prostate tissue. Since the overexpression of OPH in tumors compared to normal prostate tissue is the most ideal situation for OPH targeted prodrugs, we have limited the remainder of this study to the non-tumorigenic RWPE-1 and tumorigenic LNCaP cell lines.

### Esterase activity profiles with n-PAGE electroblotting

**Figure 5:**
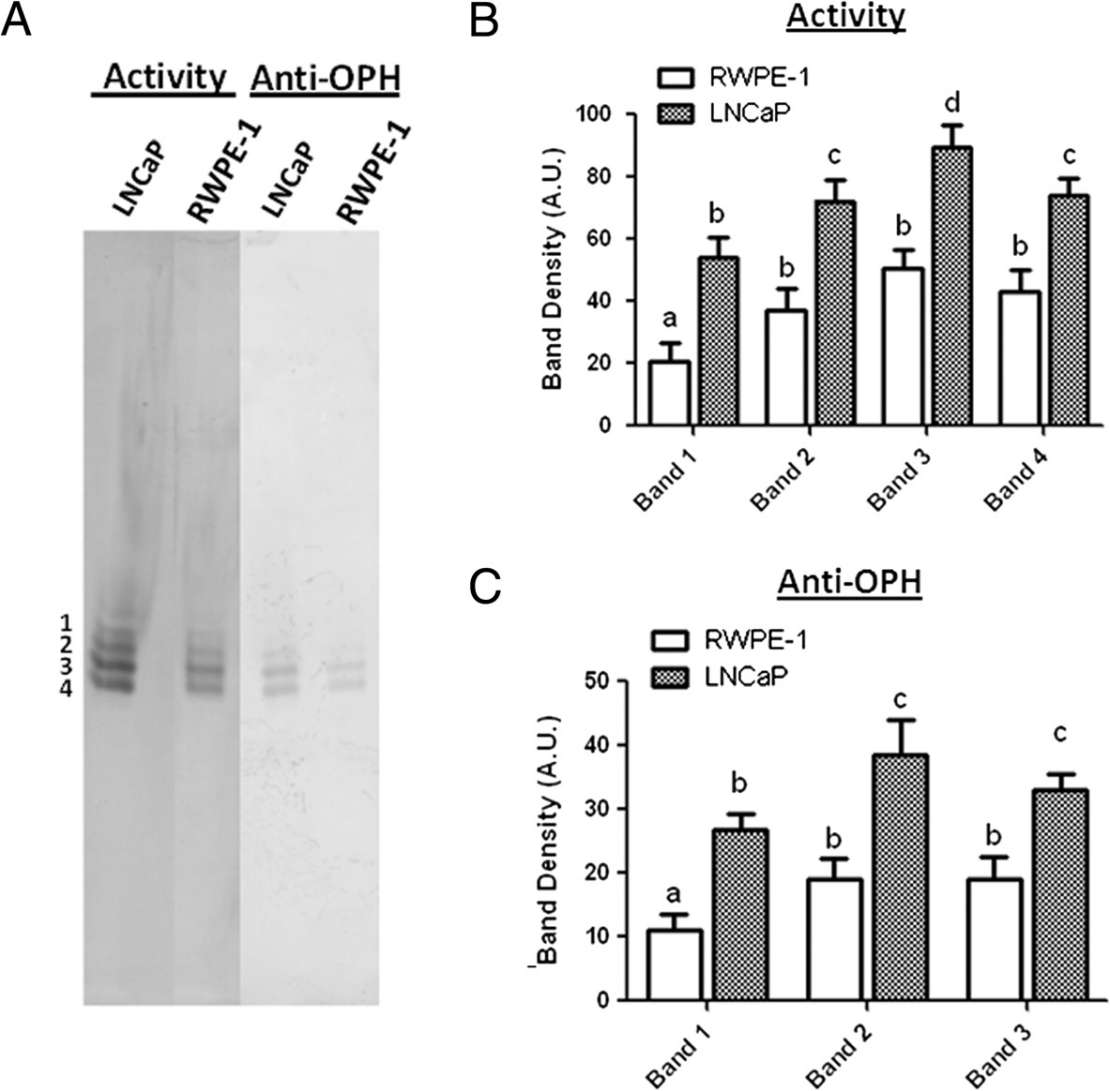
**Native electroblot activity staining and anti-OPH reveal OPH bands.** LNCaP and RWPE-1 cell lysates each containing 120 μg of protein were subjected to 6% n-PAGE followed by electroblot transfer to a nitrocellulose membrane. **A)** The blot was stained with 800 μM S-ANAA and Fast Blue RR salt. A parallel unstained blot was probed with anti-OPH as stated in the Materials and methods section. **B)** The esterase activity and **C)** anti-OPH bands were measured by densitometry and expressed in arbitrary units (A.U.). Letters that are not the same are significantly different at P < 0.05.

In order to further validate the nanospray-LC-MS/MS results we next tested the possibility that: (1) esterase activity could be maintained after n-PAGE electroblotting; (2) immunostaining could be used to confirm the presence of OPH protein in the n-PAGE esterase activity bands; (3) nanospray-LC-MS/MS could be performed on the electroblotted esterase bands. RWPE-1 and LNCaP lysates were separated on 6% n-PAGE gels and the proteins transferred to a nitrocellulose membrane by electroblotting and the esterase bands visualized by activity staining of the membrane with S-ANAA substrate. As shown in Figure 5A, this methodology resulted in the appearance of two additional sharp bands in the 220-240 kDa native protein marker region of the blot. A parallel blot was probed with anti-OPH antibody to confirm the proteomic identification of OPH within the activity bands. The esterase activity was quantified using densitometry analysis (Figure 5B) and the LNCaP activity bands showed about a 50% higher esterase activity compared to the respective RWPE-1 activity bands. Densitometry of the anti-OPH immunoblot (Figure 5C) revealed relative intensity patterns that paralleled that seen for the activity bands. The four OPH activity bands were excised separately and each band analyzed by LC/MS-MS. As detailed in Table 2, OPH was identified within the most active activity bands (bands 2-4) but could not be consistently identified within the least active band (band 1). We also noted that the esterase activity profiles with n-PAGE electroblotting had a lower level of background staining than similarly stained native gels.

**Table 2:** Electroblot esterase activity bands contain OPH


**B and 2**		
**Protein Identified**	P	MW(D a)
N-acylaminoa cly-peptide hydrolase	3.710E-05	81172.77
**Peptides Identified**	**P**	**MW(D a)**
R.QVLLSEPEEAAALYR.G	1.230E-02	556.27
K.ALDVSASDDEIAR.L	5.072E-01	103.52
K.SFNLSALEK.H	8.916E-01	159.19
K.AESFFQTK.A	9.150E-01	159.21
R.VVFDSAQR.S	8.716E-01	113.36
R.QVLLSEPEEAAALYR.G	4.985E-05	439.67
**B and 3**		
**Protein Identified**	**P**	**MW(D a)**
N-acylaminoacyl-peptide hydrolase	1.857E-07	81172.77
**Peptides Identified**	**P**	**MW(D a)**
R.QVLLSEPEEAAALYR.G	1.857E-07	1185.89
R.GSTGFGQDSILSLPGNVGHQDVK.D	3.389E-04	541.33
K.TPLLLML GQEDR.R	2.792E-05	1402.32
R.NVPVR.D	9.825E-01	91.84
**B and 4**		
**Protein Identified**	**P**	**MW(D a)**
N-acylaminoacyl-peptide hydrolase	4.579E-05	81172.77
**Peptides Identified**	**P**	**MW(D a)**
K.SPIR.Y	9.457E-01	36.07
K.SFNLSALEK.H	9.684E-01	338.72
R.QVLLSEPEEAAALYR.G	4579E-05	1022.76

LC/MS-MS of the bands excised from an activity stained native electroblot membrane confirmed the presence of OPH. OPH was not consistently identified in band 1. P indicates the probability that the protein or peptide is a random match to the spectral data.

### OPH accounts for the all the esterase staining observed with the S-ANAA substrate

**Figure 6:**
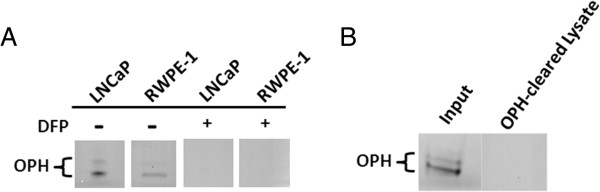
**Pre-clearance of OPH or inhibition by DFP ablates OPH activity bands. A)** LNCaP and RWPE-1 lysates were separated by 6% n-PAGE. The gel was pre-incubated in phosphate buffer or phosphate buffer containing 50 μM DFP for 30 min. Esterase activity bands were visualized with S-ANAA and Fast Blue RR salt. **B)** LNCaP lysates containing 120 μg of protein were pre-cleared with protein A beads or anti-OPH antibody bound to protein A beads. The collected lysates were separated by 6% n-PAGE and the esterase activity visualized with S-ANAA and Fast Blue RR salt.

We next investigated whether the apparent esterase activity with the S-ANAA substrate observed in the 198 and 216 kDa bands was due completely to OPH. Native-PAGE gels run with LNCaP or RWPE-1 lysates were pre-incubated with 50 μM diisopropyl fluorophosphate (DFP), a known irreversible inhibitor of serine esterases/proteases and of OPH [23], before activity staining with S-ANAA substrate (Figure 6A). The 198 kDa and 216 kDa esterase bands showed no visible activity after pre-incubation with DFP, indicating that the esterase activity observed was completely due to a serine hydrolase activity. We further confirmed this finding by pre-clearing the cell lysates with anti-OPH antibody prior to n-PAGE esterase activity profiling (Figure 6B). Activity staining with S-ANAA revealed that cell lysates pre-cleared with anti-OPH antibody had no detectable esterase activity further confirming that the esterase activity observed with S-ANAA was due specifically and completely to OPH.

### OPH present in prostate cell lysates appears as a single MW weight species in SDS-PAGE Westerns

**Figure 7:**
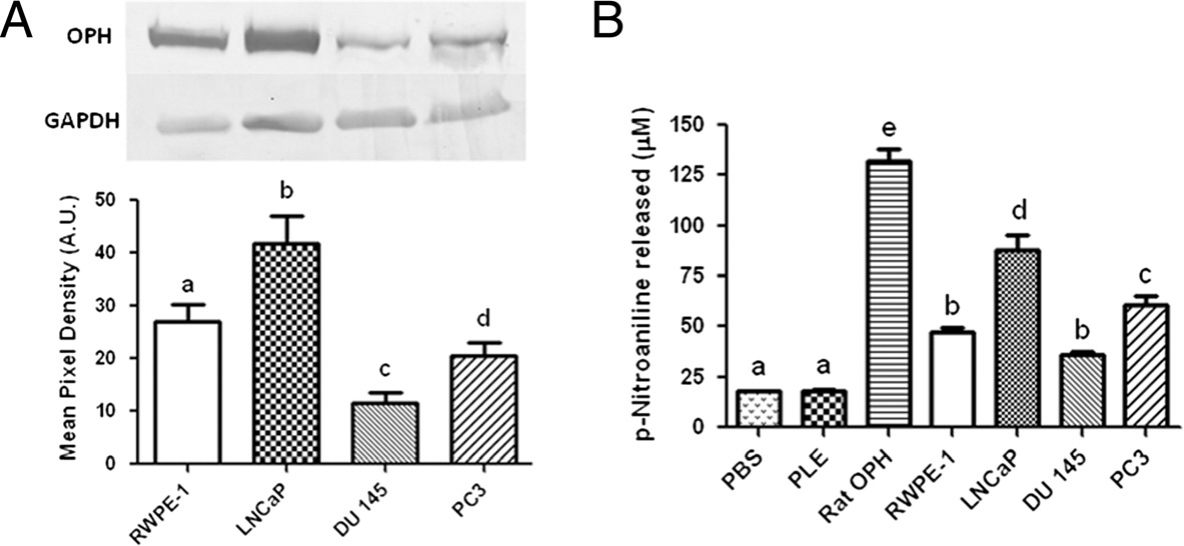
**OPH protein levels are differentially expressed in prostate cell lysates. A)** RWPE-1, LNCaP, DU 145, and PC3 lysates containing 90 μg of protein were separated by 6% SDS-PAGE, transferred to a nitrocellulose membrane, and probed with anti-OPH antibody. Anti-OPH bands were measured by densitometry and expressed in arbitrary units (A.U.). **B)** RWPE-1, LNCaP, DU 145, and PC3 lysates containing 90 μg of protein were incubated with 4 mM AcApNA and the amount of p-nitroaniline released after 10 minutes were calculated. PLE (0.5 units) and semi-purified rat liver OPH (250 ng) were used as negative and positive controls respectively. Letters that are not the same are significantly different at P < 0.05.

Since we observed multiple OPH bands in prostate cell lysates with n-PAGE, we next wanted to determine if SDS-PAGE gels similarly had multiple bands or a single OPH polypeptide with the known MW (81 kDa) of the OPH monomer. Western blots of non-tumorigenic RWPE-1 and tumorigenic LNCaP, DU 145, and PC3 cell lysates were found to be a single 81 kDa band (Figure 7A). Densitometry analysis showed significant differences in OPH expression levels among the four cell lines (Figure 7B). LNCaP cell lysates contained approximately 40% more OPH than RWPE-1, while DU 145 and PC3 lysates contained less OPH (50% and 25% respectively) than RWPE-1.

Mammalian OPH has been primarily reported as a homotetramer [16,24,25] with each OPH subunit being active within the tetramer. It has been shown that citraconylation of the amino groups of purified OPH tetramer reversibly dissociates the quaternary structure of OPH. When acylated with citraconic anhydride, OPH separated by n-PAGE forms multiple OPH bands [26]. Interestingly, our prostate cell lysates produced four uniformly distributed activity bands when separated by n-PAGE. Some explanations for the multiple OPH activity bands are OPH multi-mers, isoforms, degradation products, protein interactions, and post-translational modifications. OPH isoforms and degradation products appear to be unlikely causes for the multiple bands. Isoforms and degredation products typically result in multiple bands when separated by SDS-PAGE; however, western blots of the prostate lysates reveal a single 80kD OPH band. The interaction of native OPH with other proteins is plausible.

There is evidence that under conditions of oxidative stress OPH translocates to the cell membrane of erythrocytes and degrades oxidized proteins [27]. Similarly, OPH was found to translocate to the aggresome when the proteasome was inhibited in COS-7 cells [28]. High levels of oxidative stress are known to oxidize proteins resulting in protein aggregations that can inhibit the proteasome [29]. LNCaP, DU 145, and PC3 cell lines are reported to have significantly higher free radical production compared to RWPE-1 [30], which might induce OPH to interact with aggresomal or membrane proteins. Our mass spectrometry analysis of the OPH bands revealed several proteins known to be associated with aggresomes and were consistent with previously published data [31]. We are actively pursuing an explanation for the multiple OPH bands.

### The higher expression of OPH protein in LNCaP cell lysates is reflected by a higher activity towards N-acetyl-alanyl-p-nitroanilide

N-acetyl-alanyl-p-nitroanilide (AcApNA) (Figure 1D) is a specific OPH substrate and is routinely used to measure OPH activity and follow OPH purification from tissue homogenates [16,24,25,28]. The product p-nitroaniline (p-NA) is released upon hydrolysis of AcApNA and its absorbance measured at 405 nm. We found that non-tumorigenic and tumorigenic cell lysates incubated with AcApNA released p-NA at differential rates (Figure 7B). After 10 minutes of incubation with AcApNA, LNCaP lysates released approximately 40% more p-NA than RWPE-1 lysates. PC3 lysates released approximately 15% more p-NA compared to RWPE-1, while the rates for AcApNA hydrolysis were similar for DU 145 and RWPE-1. The activity profile of the prostate cell lysates incubated with the known OPH substrate AcApNA parallel the expression of OPH observed by SDS-PAGE Western blots (Figure 7A) as well as the esterase activity profiles observed for n-PAGE stained with S-ANAA. As expected, porcine liver esterase (PLE) had no activity towards the AcApNA substrate.

### The esterase substrates enter prostate cells and have measurable *in situ* esterase activities

**Figure 8:**
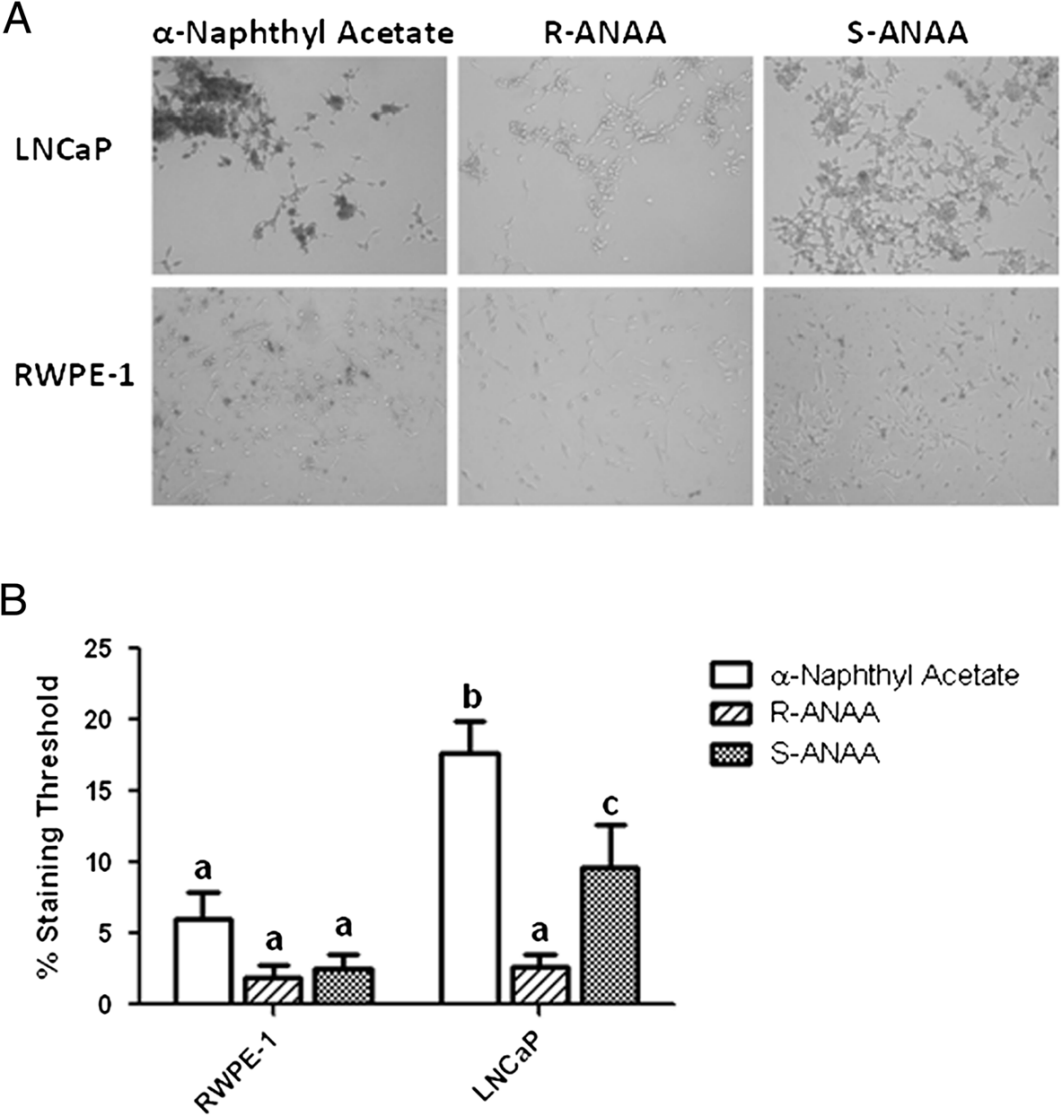
**LNCaP prostate cancer cells contain higher esterase activity than non-tumorigenic RWPE-1 cells. A)** LNCaP and RWPE-1 cell cultures were incubated with 800 μM α-naphthyl acetate, R-ANAA, or S-ANAA substrate and Fast Blue RR salt for 20 min. The dark color indicates *in situ* esterase activity. **B)** Microscopy images were analyzed with ImageJ to measure the relative staining between cell lines. Percent area threshold was defined as the percent area stained that exceeded background staining. Letters that are not the same are significantly different at P < 0.05.

As indicated in Figure 8, we next compared the esterase activities within LNCaP and RWPE-1 cultured prostate epithelial cells by incubating intact cells with α-naphthyl acetate or the chiral ANAA substrates. We found that LNCaP cells had higher *in situ* esterase activity with all three substrates compared to RWPE-1 (Figure 8A). Analyses of the areas stained (Figure 8B) showed that α-naphthyl acetate stained LNCaP cells approximately three-fold more than RWPE-1 cells. RWPE-1 cells showed no significant difference in staining between the chiral ANAA substrates; however, the LNCaP cells had a five-fold higher esterase activity level with S-ANAA compared to R-ANAA. LNCaP cells also had five-fold higher activity with S-ANAA than RWPE-1 cells. These data clearly demonstrate that the ester substrates are permeable to the plasma membrane, which is typical of neutral esters.

### Human OHP overexpressed in COS-7 has characteristics similar to that of OPH in the human prostate epithelial cell lines

**Figure 9:**
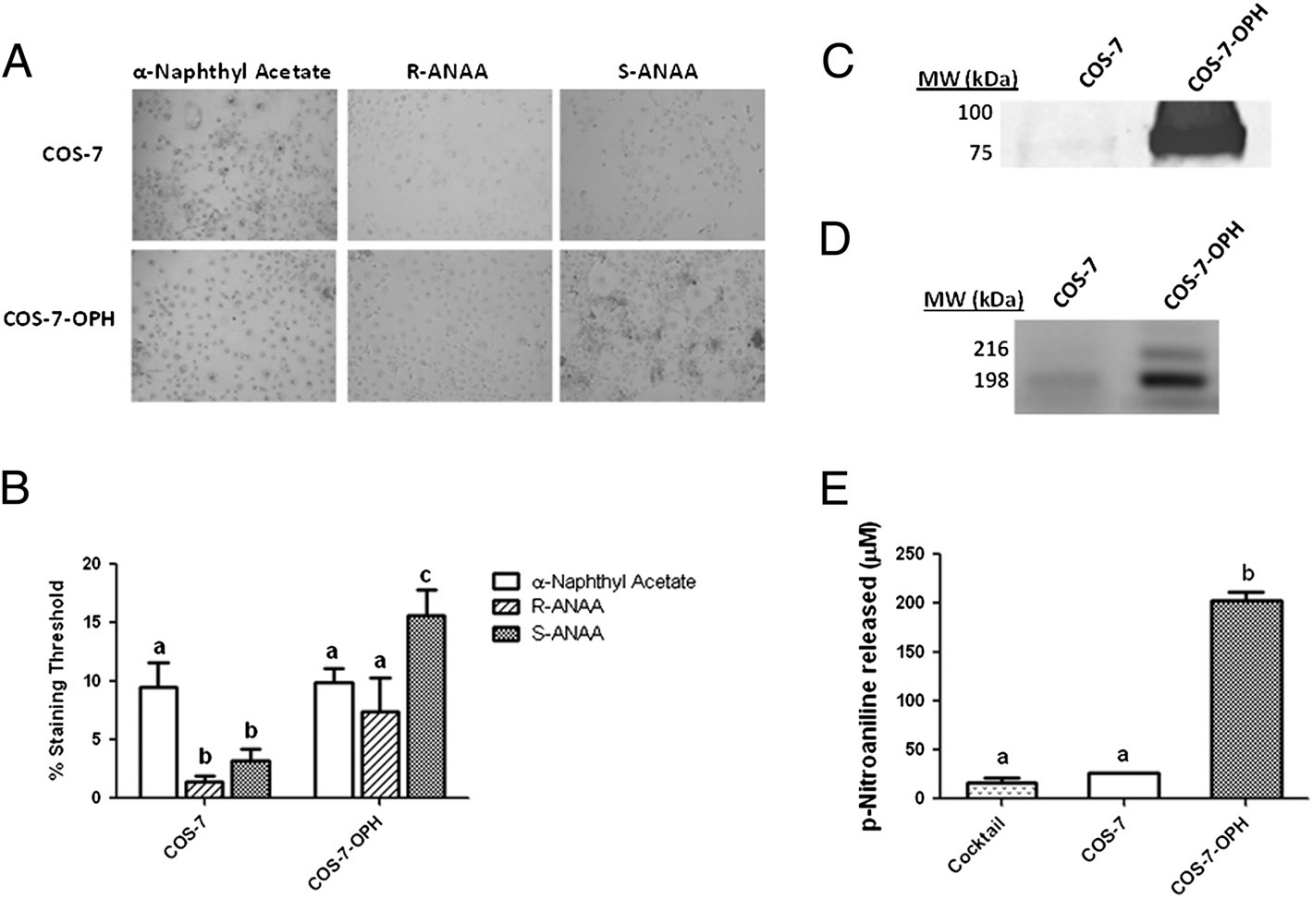
**COS-7 cells that over-express OPH show higher levels of ANAA hydrolysis. A)** COS-7 and COS-7-OPH cells were treated with 800 μM α-naphthyl acetate, R-ANAA, or S-ANAA substrate and Fast Blue RR salt for 20 minutes. Dark color indicates esterase activity. **B)** Microscopy images were analyzed with ImageJ to measure the relative staining between cell lines. Percent area threshold was defined as the percent area stained that exceeded background staining. **C)** Anti-OPH Western blot of COS-7 and COS-7-OPH lystates containing 90 μg of total protein. **D)** Cell lysates containing 120 μg of protein were separated by 6% n-PAGE followed by staining with 800 μM S-ANAA. **E)** Lysates were incubated with 4 mM N-acetyl-L-alanyl-p-nitroanilide and the amounts of p-nitroanaline released after 10 minutes were measured. Letters that are not the same are significantly different at P < 0.05.

As a positive control, we next repeated the *in situ* experiment with COS-7 cells and COS-7-OPH cells which overexpress OPH (Figure 9A). As expected, based on our n-PAGE experiments (Figure 4A), there was no increase in COS-7-OPH with *in situ* staining when α-naphthyl acetate was used since it was not found to be a substrate for OPH. However, there were significant increases in esterase activity staining with the chiral ANAA substrates. There was approximately a seven-fold increase in esterase activity staining with S-ANAA in the COS-7-OPH cells compared to the non-transfected COS-7 cells. Moreover, there was approximately 50% more esterase activity staining with the S-isomer compared to the R-isomer. As indicated in Figure 9C, SDS-PAGE Western blots of COS-7 and COS-7-OPH lysates using anti-OPH antibody confirms the marked overexpression of OPH in the COS-7-OPH cells. Additionally, n-PAGE activity profiling with S-ANAA and the OPH activity assay confirms the overexpression of active OPH in the COS-7-OPH cells and also show the OPH activity is present in two main bands (Figure 9D). Lysates of COS-7 and COS-7-OPH were incubated with AcApNA for 10 min and the p-NA released was compared (Figure 9E). COS-7-OPH lysates showed approximately a seven-fold higher p-NA release compared to COS-7 lysates.

## Discussion and Conclusion

A number of investigators have suggested that chiral ester prodrugs hold the promise of providing more selective anticancer chemotherapy [7-9,11,14]. A major requirement for this strategy is the need to identify target esterases that have differential expression or substrate selectivity in cancer cells compared to their normal counterpart. Ideally, esterases targeted for prodrug hydrolysis should be highly expressed in the target tumor cells and/or have a chiral preference different from normal cells. Yamazaki et al. previously found that several cancers displayed hydrolytic preferences for isomers of chiral substrates opposite that of their normal counterparts [13,14,32]. However, in the work presented here we found that the esterases of both tumorigenic and non-tumorigenic prostate cells both showed a preference for the S-isomer of α-naphthyl N-acetyl-alaninate (S-ANAA).

Additionally, we have improved upon the work by Yamazaki et al. by identifying a specific esterase that has differential activity towards chiral ANAA substrates. We have used several proteomic techniques to identify OPH in tumorigenic and non-tumorigenic prostate cells. Using an n-PAGE method similar to Yamazaki et al., n-PAGE electroblotting, immunoblotting, inhibition studies and mass spectrometry we have identified OPH in prostate cells and have found that OPH has selective activity towards chiral ANAA substrates.

OPH is a serine protease and a member of the prolyl oligopeptidase (POP) family. Three functions of OPH have been described: (1) an exopeptidase activity that unblocks N-acetyl peptides with a preference for N-acetyl alanyl peptides [16]; (2) an endopeptidase activity towards oxidized and glycated proteins [28,33-36] and; (3) an ability to associate with aggresomes when proteasome function is inhibited [28]. Moreover, work by Shimizu et al. [28] suggests that the proteasome and OPH work coordinately to clear cells of oxidized (carbonylated) proteins. A comprehensive physiological understanding of OPH remains elusive. Nevertheless, the acetylation of the N-terminal α-amine group of proteins is the most common post-translational modification in eukaryotic proteins yet, little is known about the biological role of N-alpha-terminal acetylation, and even less is known about the role of enzymes (like OPH) that catalyze the hydrolysis of an N-terminally acetylated peptide to release an N-acetylamino acid.

Our finding that OPH in non-tumorigenic and tumorigenic prostate cell lines have a greater hydrolytic preference for the S-ANAA isomer of ANAA is consistent with previous observations that OPH has a preference for small peptides with Ac-L-alanine (Ac-S-alanine) compared to Ac-D-alanine (Ac-R-alanine) [37]. OPH activity bands were not observed with the α-naphthyl acetate substrate while distinct activity bands were visualized using α-naphthyl N-acetyl-alaninate substrates. In addition, OPH has high specificity for AcApNA and Ac-Ala-β-naphthylamide [16]. AcApNA and Ac-Ala-β-naphthylamide are structurally similar to ANAA; the main differences being the 4-nitrobenzene of AcApNA and the peptide bond of Ac-Ala-β-naphthylamide. AcApNA, Ac-Ala-β-naphthylamide, and ANAA contain N-terminal acetylated alanine. Taken together, this family of small N-acetyl-alaninate substrates appears to be good models for future OPH targeted prodrug designs.

We found OPH to be differentially expressed in the LNCaP prostate cancer cell line compared to non-tumorigenic RWPE-1 cell lines and that tumor cells overexpressing OPH might be responsive to prodrug derivatives of α-naphthyl N-acetyl-alaninate. We have found that OPH expression in prostate cancer cells can vary widely. DU-145 and PC3 lysates showed slightly diminished levels of OPH compared to RWPE-1 while LNCaP contained nearly twofold more OPH than RWPE-1. OPH protein expression levels appear to vary in other cancers as well. Serine protease activity profiling performed by Jessani et al. shows that OPH is highly active in several melanoma and breast cancer cell lines [38]; however, Scaloni previously reported that OPH was deleted or under-expressed in a number of small cell lung carcinomas [37]. We have screened other tumorigenic cell lines for OPH activity and have found that several aggressive colon cancer and melanoma cell lines exhibit significantly higher OPH activity compared to their non-tumorigenic counterparts. Further OPH expression profiling of normal prostate cell lines, prostate cancer cell lines, and primary prostate tissues is needed to determine which prostate cancers might be suitable for OPH-targeted therapies.

OPH has recently been proposed as a potential target for the development of anticancer drugs [39]. Histological data in the Human Protein Atlas shows that OPH can be strongly expressed in some cases of colorectal, breast, prostate, ovarian, endometrial and liver cancers [40]. OPH has a well-documented substrate specificity towards N-acetylated-L-alaninate esters [41]. Our results suggest that α-naphthyl N-acetyl-alaninate substrates could be used to rapidly determine levels of active OPH in non-tumorigenic and tumorigenic cells and tissues. Naphthyl substrates are routinely used to differentiate chronic myelogenous leukemia (CML) from leukemoid reaction and to distinguish other myeloproliferative disorders [42,43]. Yamazaki et al. demonstrated that a substrate similar to S-ANAA, N-methoxycarbonylalaninate, could be used to visualize esterase activity in cryostat tissue sections [32]. Our cell culture activity staining of RWPE-1, LNCaP, and COS-7-OPH cells with S-ANAA suggest that S-ANAA may be useful to screen cells and tissues for relative OPH activity. Screening normal and tumorigenic cells or tissues with S-ANAA may aid in identifying candidates for OPH directed therapies.

In conclusion, we have found that cell lysates from non-tumorigenic RWPE-1 cells and several tumorigenic prostate cell lines display differential esterase activity profiles when visualized with α-naphthyl acetate or chiral α-naphthyl N-acetyl-alaninate substrates. Our n-PAGE results show that tumorigenic LNCaP, DU 145, and PC3 cell lysates contain higher general esterase activity when visualized with α-naphthyl acetate compared to non-tumorigenic RWPE-1 cell lysates. In addition, we found that the OPH activity of prostate cell lysates could be visualized by staining with chiral α-naphthyl N-acetyl-alaninate substrates, and that OPH has a hydrolytic preference for the S-isomer of ANAA. LNCaP lysates in particular showed the highest esterase activity with all of the ester substrates tested and contained the highest OPH activity measured with AcApNA. The results of this study indicate that ester prodrugs designed after S-ANAA or AcApNA may be a promising therapeutic approach to prostate cancers that overexpress OPH.

## Competing interests

The authors declare that they have no competing interests.

## Authors’ contributions

CM, YJ, VP, MB, KK, and WS conceived and designed studies, CM, YJ, VP, and MB acquired and analyzed data, and CM and WS wrote the manuscript. All authors read, edited, and approved the final manuscript.

## Pre-publication history

The pre-publication history for this paper can be accessed here:

http://www.biomedcentral.com/1471-2407/14/77/prepub

## References

[B1] American Cancer Society Cancer Facts & Figures 2013 2013 Atlanta: American Cancer Society

[B2] Bodey GP Rodriguez V McCredie KB Freireich EJ Neutropenia and infection following cancer chemotherapy Int J Radiat Oncol Biol Phys 1976 1 3–4 301 304 972090 10.1016/0360-3016(76)90056-0

[B3] Deinard AS Fortuny IE Theologides A Anderson GL Boen J Kennedy BJ Studies on the neutropenia of cancer chemotherapy Cancer 1974 33 5 1210 1218 10.1002/1097-0142(197405)33:5<1210::AID-CNCR2820330503>3.0.CO;2-Q 4595975

[B4] Dearnaley DP Khoo VS Norman AR Meyer L Nahum A Tait D Yarnold J Horwich A Comparison of radiation side-effects of conformal and conventional radiotherapy in prostate cancer: a randomised trial Lancet 1999 353 9149 267 272 10.1016/S0140-6736(98)05180-0 9929018

[B5] Randall J Ream E Hair loss with chemotherapy: at a loss over its management? Eur J Canc Care (Engl) 2005 14 3 223 231 10.1111/j.1365-2354.2005.00558.x 15952966

[B6] Richardson A Ream E Wilson-Barnett J Fatigue in patients receiving chemotherapy: patterns of change Cancer Nurs 1998 21 1 17 30 10.1097/00002820-199802000-00003 9494227

[B7] Connors TA Whisson ME Cure of mice bearing advanced plasma cell tumours with aniline mustard: the relationship between glucuronidase activity and tumour sensitivity Nature 1966 210 5038 866 867 5958471 10.1038/210866b0

[B8] Carl PL Chakravarty PK Katzenellenbogen JA Weber MJ Protease-activated "prodrugs" for cancer chemotherapy Proc Natl Acad Sci U S A 1980 77 4 2224 2228 10.1073/pnas.77.4.2224 6246527 PMC348685

[B9] Watanabe KA Matsuda A Halat MJ Hollenberg DH Nisselbaum JS Fox JJ Nucleosides. 114. 5'-O-Glucuronides of 5-fluorouridine and 5-fluorocytidine. Masked precursors of anticancer nucleosides J Med Chem 1981 24 7 893 897 10.1021/jm00139a026 7277401

[B10] Kageyama Y Yamazaki Y Okuno H Novel approaches to prodrugs of anticancer diaminodichloroplatinum(II) complexes activated by stereoselective enzymatic ester hydrolysis J Inorg Biochem 1998 70 1 25 32 10.1016/S0162-0134(98)00009-9 9661285

[B11] Bardos TJ Chmielewicz ZF Hebborn P Structure-activity relationships of alkylating agents in cancer chemotherapy Ann N Y Acad Sci 1969 163 2 1006 1025

[B12] Yamazaki Y Furukawa F Nishikawa A Takahashi M Oka S Histochemical determination of stereoselectivity of esterases in normal pancreas and pancreatic tubular adenocarcinoma of hamsters Biotech Histochem 1998 73 1 23 31 10.3109/10520299809140503 9554581

[B13] Yamazaki Y Kageyama Y Okuno H Direct evaluation of stereoselectivity of cancer esterases by polyacrylamide gel electrophoresis coupled with activity staining with chiral naphthyl esters Anal Biochem 1995 231 2 295 300 10.1006/abio.1995.9996 8594976

[B14] Yamazaki Y Ogawa Y Afify AS Kageyama Y Okada T Okuno H Yoshii Y Nose T Difference between cancer cells and the corresponding normal tissue in view of stereoselective hydrolysis of synthetic esters Biochim Biophys Acta 1995 1243 3 300 308 10.1016/0304-4165(94)00153-O 7727503

[B15] Jones WM Scaloni A Bossa F Popowicz AM Schneewind O Manning JM Genetic relationship between acylpeptide hydrolase and acylase, two hydrolytic enzymes with similar binding but different catalytic specificities Proc Natl Acad Sci U S A 1991 88 6 2194 2198 10.1073/pnas.88.6.2194 2006156 PMC51196

[B16] Kobayashi K Smith JA Acyl-peptide hydrolase from rat liver. Characterization of enzyme reaction J Biol Chem 1987 262 24 11435 11445 3305492

[B17] Perrier J Durand A Giardina T Puigserver A Catabolism of intracellular N-terminal acetylated proteins: involvement of acylpeptide hydrolase and acylase Biochimie 2005 87 8 673 685 10.1016/j.biochi.2005.04.002 15927344

[B18] Koltun D Parkhill E Boze M Zablocki J Vasilevich N Mayboroda E Glushkov A Cole AG Chisholm J Preparation of 3-hydroquinazolin-4-one derivatives and analogs thereof for use as stearoyl CoA desaturase inhibitors. *U.S. Pat. Appl. Publ.* (2009), US 20090105283 A1 20090423 Chem Abstract 2008 149 471495

[B19] Broemme D Bescherer K Kirschke H Fittkau S Enzyme-substrate interactions in the hydrolysis of peptides by cathepsins B and H from rat liver Biochem J 1987 245 2 381 385 3663163 10.1042/bj2450381 PMC1148133

[B20] Tsunasawa S Narita K Ogata K Purification and properties of acylamino acid-releasing enzyme from rat liver J Biochem 1975 77 1 89 102 1137989

[B21] Muller J Keller HU Durig P Hagmann J Cornioley DM Reinhard J Ruchti C Hess MW Cottier H Nonspecific esterase in human lymphocytes Int Arch Allergy Appl Immunol 1981 64 4 410 421 10.1159/000232721 6162799

[B22] Knowles DM Hoffman T Ferrarini M Kunkel HG The demonstration of acid alpha-naphthyl acetate esterase activity in human lymphocytes: usefulness as a T-cell marker Cell Immunol 1978 35 1 112 123 10.1016/0008-8749(78)90131-4 304379

[B23] Miyagi M Sakiyama F Kato I Tsunasawa S Complete covalent structure of porcine liver acylamino acid-releasing enzyme and identification of its active site serine residue J Biochem 1995 118 4 771 779 8576092 10.1093/oxfordjournals.jbchem.a124979

[B24] Sharma KK Ortwerth BJ Bovine lens acylpeptide hydrolase. Purification and characterization of a tetrameric enzyme resistant to urea denaturation and proteolytic inactivation Eur J Biochem 1993 216 2 631 637 10.1111/j.1432-1033.1993.tb18183.x 8375399

[B25] Scaloni A Barra D Jones WM Manning JM Human acylpeptide hydrolase. Studies on its thiol groups and mechanism of action J Biol Chem 1994 269 21 15076 15084 8195144

[B26] Durand A Villard C Giardina T Perrier J Juge N Puigserver A Structural properties of porcine intestine acylpeptide hydrolase J Protein Chem 2003 22 2 183 191 10.1023/A:1023431215558 12760423

[B27] Fujino T Ando K Beppu M Kikugawa K Enzymatic removal of oxidized protein aggregates from erythrocyte membranes J Biochem 2000 127 6 1081 1086 10.1093/oxfordjournals.jbchem.a022701 10833278

[B28] Shimizu K Kiuchi Y Ando K Hayakawa M Kikugawa K Coordination of oxidized protein hydrolase and the proteasome in the clearance of cytotoxic denatured proteins Biochem Biophys Res Commun 2004 324 1 140 146 10.1016/j.bbrc.2004.08.231 15464994

[B29] Davies KJ Degradation of oxidized proteins by the 20S proteasome Biochimie 2001 83 3–4 301 310 11295490 10.1016/s0300-9084(01)01250-0

[B30] Kumar B Koul S Khandrika L Meacham RB Koul HK Oxidative stress is inherent in prostate cancer cells and is required for aggressive phenotype Cancer Res 2008 68 6 1777 1785 10.1158/0008-5472.CAN-07-5259 18339858

[B31] Pallotta V D'Alessandro A Rinalducci S Zolla L Native protein complexes in the cytoplasm of red blood cells J Proteome Res 2013 12 7 3529 3546 10.1021/pr400431b 23781972

[B32] Yamazaki Y Oka S In situ evaluation of esterase stereoselectivity in two-dimensional electropherograms and tissue sections J Chromatogr B Biomed Sci Appl 1998 709 2 306 309 10.1016/S0378-4347(98)00073-5 9657229

[B33] Harmat V Domokos K Menyhard DK Pallo A Szeltner Z Szamosi I Beke-Somfai T Naray-Szabo G Polgar L Structure and catalysis of acylaminoacyl peptidase: closed and open subunits of a dimer oligopeptidase J Biol Chem 2011 286 3 1987 1998 10.1074/jbc.M110.169862 21084296 PMC3023495

[B34] Nakai A Yamauchi Y Sumi S Tanaka K Role of acylamino acid-releasing enzyme/oxidized protein hydrolase in sustaining homeostasis of the cytoplasmic antioxidative system Planta 2012 236 2 427 436 10.1007/s00425-012-1614-1 22398639

[B35] Shimizu K Ikegami-Kawai M Takahashi T Increased oxidized protein hydrolase activity in serum and urine of diabetic rat models Biol Pharm Bull 2009 32 9 1632 1635 10.1248/bpb.32.1632 19721247

[B36] Kikugawa K Defense of living body against oxidative damage Yakugaku Zasshi 2004 124 10 653 666 10.1248/yakushi.124.653 15467273

[B37] Scaloni A Jones W Pospischil M Sassa S Schneewind O Popowicz AM Bossa F Graziano SL Manning JM Deficiency of acylpeptide hydrolase in small-cell lung carcinoma cell lines J Lab Clin Med 1992 120 4 546 552 1328432

[B38] Jessani N Liu Y Humphrey M Cravatt BF Enzyme activity profiles of the secreted and membrane proteome that depict cancer cell invasiveness Proc Natl Acad Sci U S A 2002 99 16 10335 10340 10.1073/pnas.162187599 12149457 PMC124915

[B39] Palmieri G Bergamo P Luini A Ruvo M Gogliettino M Langella E Saviano M Hegde RN Sandomenico A Rossi M Acylpeptide hydrolase inhibition as targeted strategy to induce proteasomal down-regulation PLoS One 2011 6 10 e25888 10.1371/journal.pone.0025888 22016782 PMC3189933

[B40] Uhlen M Oksvold P Fagerberg L Lundberg E Jonasson K Forsberg M Zwahlen M Kampf C Wester K Hober S Wernerus H Bjorling L Ponten F Towards a knowledge-based human protein atlas Nat Biotechnol 2010 28 12 1248 1250 10.1038/nbt1210-1248 21139605

[B41] Gade W Brown JL Purification and partial characterization of alpha-N-acylpeptide hydrolase from bovine liver J Biol Chem 1978 253 14 5012 5018 670173

[B42] KAPLOW LS A histochemical procedure for localizing and evaluating leukocyte alkaline phosphatase activity in smears of blood and marrow Blood 1955 10 10 1023 1029 13260361

[B43] Lippi U Cappelletti P Schinella M Alpha-naphthyl butyrate esterase (a selective cytochemical monocyte marker) Ric Clin Lab 1983 13 4 467 471 6658305

